# Carboxy terminus of GATA4 transcription factor is required for its cardiogenic activity and interaction with CDK4

**DOI:** 10.1016/j.mod.2014.09.001

**Published:** 2014-11

**Authors:** Joseph M. Gallagher, Abir Yamak, Pavel Kirilenko, Sarah Black, Matthias Bochtler, Chantal Lefebvre, Mona Nemer, Branko V. Latinkić

**Affiliations:** a School of Biosciences, Cardiff University, Cardiff, Wales, United Kingdom; b Laboratory of Cardiac Development and Differentiation, Department of Biochemistry, Microbiology, and Immunology, University of Ottawa, Ottawa, Ontario, Canada; c International Institute of Molecular and Cellular Biology, Warsaw, Poland; d Department of Bioinformatics, Institute of Biochemistry and Biophysics, Warsaw, Poland

**Keywords:** GATA4, Cardiogenesis, CDK4

## Abstract

GATA4-6 transcription factors regulate numerous aspects of development and homeostasis in multiple tissues of mesodermal and endodermal origin. In the heart, the best studied of these factors, GATA4, has multiple distinct roles in cardiac specification, differentiation, morphogenesis, hypertrophy and survival. To improve understanding of how GATA4 achieves its numerous roles in the heart, here we have focused on the carboxy-terminal domain and the residues required for interaction with cofactors FOG2 and Tbx5. We present evidence that the carboxy terminal region composed of amino acids 362–400 is essential for mediating cardiogenesis in *Xenopus* pluripotent explants and embryos. In contrast, the same region is not required for endoderm-inducing activity of GATA4. Further evidence is presented that the carboxy terminal cardiogenic region of GATA4 does not operate as a generic transcriptional activator. Potential mechanism of action of the carboxy terminal end of GATA4 is provided by the results showing physical and functional interaction with CDK4, including the enhancement of cardiogenic activity of GATA4 by CDK4. These results establish CDK4 as a GATA4 partner in cardiogenesis. The interactions of GATA4 with its other well described cofactors Tbx5 and FOG2 are known to be involved in heart morphogenesis, but their requirement for cardiac differentiation is unknown. We report that the mutations that disrupt interactions of GATA4 with Tbx5 and FOG2, G295S and V217G, respectively, do not impair cardiogenic activity of GATA4. These findings add support to the view that distinct roles of GATA4 in the heart are mediated by different determinants of the protein. Finally, we show that the rat GATA4 likely induces cardiogenesis cell autonomously or directly as it does not require activity of endodermal transcription factor Sox17, a GATA4 target gene that induces cardiogenesis non-cell autonomously.

## Highlights

• Carboxy terminal region of GATA4 is required for cardiogenesis in *Xenopus* pluripotent explants and in embryos.
• Carboxy terminus of GATA4 interacts with CDK4.
• CDK4 enhances transcriptional and cardiogenic activity of GATA4.
• GATA4-Tbx5 and GATA4-FOG2 interactions are not required for cardiogenesis.

## 1 Introduction

GATA family of Zn finger transcription factors constitutes 6 multifunctional regulators of numerous aspects of vertebrate embryonic development and adult physiology. GATA4-6 factors are preferentially involved in development and function of mesodermal (heart, vasculature, gonads) and endodermal organs (lungs, liver, gut).

Perhaps the best studied among the GATA4-6 factors is GATA4, and in particular its roles in cardiovascular development and homeostasis. Molecular genetic analyses in the mouse, zebrafish and *Xenopus*, as well as information from patients, have revealed specific roles of GATA4 in virtually all stages and all aspects of heart development and postnatal function – specification, differentiation, growth, morphogenesis, regeneration, cell survival and hypertrophy (Aries et al, 2004, Charron et al, 2001, Garg et al, 2003, Haworth et al, 2008, Holtzinger, Evans, 2005, Kikuchi et al, 2010, Liang et al, 2001a, Liang et al, 2001b, Pu et al, 2004, Watt et al, 2004).

Together with chromatin remodelling factor BAF60c and Tbx5, GATA4 can induce ectopic cardiogenesis in the mouse embryo (Takeuchi and Bruneau, 2009), whereas in the *Xenopus* embryo and in P19 cells it is sufficient on its own (Grepin et al, 1997, Latinkic et al, 2003). GATA4 is an essential component of various cocktails of transcription factors that have been shown to reprogram non-cardiac somatic cells into cardiomyocytes (Fu et al, 2013, Ieda et al, 2010, Nam et al, 2013, Qian et al, 2012).

In the adult mammalian heart, GATA4 mediates cardiomyocyte hypertrophy in a process that is thought to resemble aspects of cardiac development, as suggested by activation of a set of cardiomyocyte gene products that are normally expressed in developing, immature cells in cells undergoing hypertrophy (Frey, Olson, 2003, Molkentin, Dorn, 2001).

However, the roles of GATA4 in development versus hypertrophy are clearly distinct, as shown by the finding that Ser 105 of GATA4, whose phosphorylation by MAPK is required for hypertrophy, is not involved in mediating cardiogenic activity of GATA4 and is not required for heart development (Gallagher et al, 2012, van Berlo et al, 2011).

GATA4 is thought to achieve its roles through a multitude of mechanisms, including regulated expression, participation in various multiprotein complexes and extensive post-translational modifications (Zhou et al., 2012). However, the roles that these modifications play in mediating various activities of GATA4 *in vivo* are just beginning to emerge. One example is the role of MAPK-mediated phosphorylation of S105 in mediating cardiac hypertrophy (van Berlo et al., 2011). Another one is provided by a recent finding that Cyclin D2 is a cofactor of GATA4 in cardiogenesis (Yamak et al., 2014). The physical and functional interactions between GATA4 and Cyclin D2 depend on phosphorylation of Ser 160 of GATA4, which can be mediated *in vitro* by CDK4 (Yamak et al., 2014).

The physiological roles for interactions of transcription factor partners of GATA4 have been studied for Tbx5 and FOG2. The GATA4-FOG2 interaction was investigated using homozygous mice carrying an interaction-disrupting V217G mutation; these animals die in mid-gestation due to the absence of coronary blood vessels and suffer defective morphogenesis of the valves and major blood vessels (Crispino et al., 2001). This phenotype is distinct from the null GATA4 and myocardium-specific knockouts, but is similar to the FOG2 null phenotype (Tevosian et al., 2000), establishing a specific role for GATA4-FOG2 interaction in the development of coronary blood vessels. Another example of a physiologically important partnering of GATA4 was provided by the finding that the G296S mutation affects the interaction between GATA4 and Tbx5 and it leads to congenital heart disease in patients (Garg et al., 2003), while in homozygous mutant mice it causes mid-gestation lethality (Misra et al., 2012).

To improve our knowledge of the mechanisms through which GATA4 acts in heart development, we focused on the roles played by the C-terminus and the residues important for the interaction with Tbx5 and FOG2 in GATA4-induced cardiac differentiation.

## 2 Material and methods

### 2.1 *Xenopus* embryos and explants

This work was approved by Cardiff University's Ethical Review Committee and was carried out under a license from the UK Home Office. *Xenopus* embryos were obtained and cultured as previously described (Sive et al., 2000). Explants (stage 8.5) were dissected and cultured in 75% Normal Amphibian Media (NAM); supplemented with gentamycin sulphate (50 µg/ml; Sigma). All rat GATA4 plasmids were linearised with Asp718 (Roche). The wt and 201–440 rGATA4 plasmids have been previously described (Gallagher et al., 2012). *Xenopus tropicalis* CDK4 full-length coding sequence in pCS107 (TGas100g18; Gilchrist et al., 2004) and Cyclin D2 (Yamak et al., 2014) were used as a template to make capped RNA. Sox17β and Sox17β::EnR transcription templates were generated as described (Hudson et al., 1997). Capped mRNA was synthesised using mMessageMachine kit (Ambion/ABI), and RNA was subsequently purified on Sephadex G50 columns (ProbeQuant; GE Healthcare). RNA samples were injected into embryos using an IM 300 Micro-injector (Narishige Scientific). For animal cap explant experiments, 400 pg of GATA4 RNA was co-injected with a mix of rhodamine-dextran (20 mg/ml) and dextran-biotin (25 mg/ml) lineage tracers (Invitrogen) (Latinkic et al., 2003) in 10 nl/embryo at 1–2 cell-stage. All GATA4 variants were tested at least twice and most constructs, including non-cardiogenic 1–361, were tested more than four times. For ectopic cardiac tissue formation assay, 2 nl (80 pg) of RNA was injected in one anterior dorsal blastomere at 8–16 cell stage. These ectodermal precursors are away from the developing heart and are permissive for ectopic cardiac tissue induction by GATA4 (Gallagher et al., 2012). In this assay, 10–15% of embryos injected with cardiogenic GATA4 variants typically show ectopic expression; total number of embryos examined was n = 40–60 from two or three independent experiments. Given relatively low penetrance of ectopic cardiogenesis in this assay, but unequivocal nature of induction of ectopic cardiac expression, the results are qualitative and conclusive when n ≥ 40. 200 pg of Sox17β or Sox17β::EnR capped mRNA was injected per embryo (Fig. 6). Cardiac actin-GFP transgenic line and its use in detecting cardiogenesis in animal pole explants were described (Latinkic et al, 2002, Latinkic et al, 2003).

### 2.2 Cell cultures and transfections

National Institutes of Health NIH3T3 cells lines were maintained and transfected as previously described (Yamak et al., 2014). The CDK inhibitor RO506220 was obtained from Roche (Burgess et al, 2006, Yamak et al, 2014). All plasmids used were previously described (Yamak et al., 2014).

### 2.3 Recombinant DNA

For gain of function studies in *Xenopus*, rat GATA4 sequences together with an N-terminal HA tag were generated by high fidelity PCR using Phusion polymerase (NEB) and were cloned in pCS2 vector via BamHI/EcoRI sites. Primer sequences are available upon request. Two copies of the minimal transcriptional activation domain of VP16 (amino acids 413–454) together with the flexible hinge region of the lambda repressor (Brickman et al., 2000) were fused to rGATA4 1–361 and 201–440 at the C-terminal end by overlap PCR. Expected coding regions generated by PCR were confirmed by sequencing.

### 2.4 Reverse Transcription-PCR (RT-PCR)

Total RNA was isolated from samples using the acid guanidinium thiocyanate-phenol-chloroform method (Chomczynski and Sacchi, 1987). Approx. 20 animal caps were used per sample, and cDNA was synthesised using MMLV-Reverse Transcriptase and random hexamers (both Invitrogen) according to the manufacturer's instructions. Primer sequences and PCR cycling conditions were as described (Samuel and Latinkic, 2009). PCR was performed with GoTaq enzyme (Promega).

### 2.5 Whole-mount in situ hybridisation (WMISH)

WMISH was carried out as described (Sive et al., 2000), using the digoxigenin (Roche)-labelled antisense riboprobe for myl7 (also known as MLC2; Chambers et al., 1994). Colour was developed using BM Purple (Roche).

### 2.6 Protein analyses

Immunohistochemistry on blastula-stage *Xenopus* embryos was performed as described (Sive et al., 2000), using rat monoclonal α-HA-HRP (1:2,500; Roche) and DAB substrate (Sigma). Images were taken on Leica MZ16 stereomicroscope with DFC300FX Leica camera and were processed with Adobe Photoshop software. Western blotting of *Xenopus* protein extracts was performed as described (8). Total cell extracts were prepared from 1–2 µl lysis buffer/animal cap. 10 µl samples were resolved by polyacrylamide gel electrophoresis and transferred to PVDF membrane (Millipore) and blotted by standard techniques. Antibodies used were rat monoclonal α-HA-HRP (1:2,500; Roche) and as a loading control α-Erk (1:2,500; Santa-Cruz). Secondary antibody was goat α-rabbit HRP (Chemicon; 1:5,000). Detection was achieved using chemiluminescent detection kit (Thermo/Pierce). For *in vitro* pull-down assay, the recombinant GST-GATA4 proteins production and pull down assays were performed as described previously (Yamak et al., 2014). For luciferase assays *Xenopus* embryos were injected with indicated RNAs together with 30 pg each of 2XGATALuc (Grepin et al., 1994) and TK-RL (Promega). Dual Luciferase Assays (Promega) were carried out as recommended by the manufacturer on extracts from approximately 20 animal caps per sample, collected 3 hours after excision at st. 9.

### 2.7 Molecular modelling

Modelling was carried out using SWISSMODEL (Arnold et al., 2006). The figure was created with PyMOL software (http://www.pymol.org).

## 3 Results

### 3.1 Carboxy-terminal domain of GATA4 is required for cardiogenesis

**Fig. 1:**
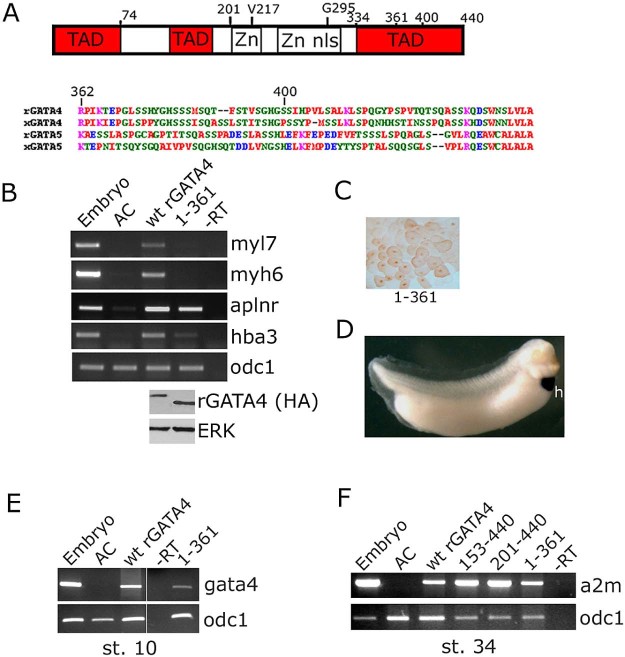
Carboxy-terminal domain (362–440) of GATA4 is required for cardiogenesis. (A) Schematic representation of GATA4 structure showing transcriptional activation domains (TAD), Zn fingers (Zn), nuclear localisation signal (nls) and the position of mutations tested in this study. Right – sequence of the C-terminal region of GATA4 (rat, NP_653331; *Xenopus*NP_001085355) and GATA5 (rat NP_001019487; *Xenopus*NP_001081962), showing poor conservation in these cardiogenic factors. (B) Deletion of C-terminal 78 amino acids abolishes cardiogenic activity of GATA4. Animal caps injected with wt or 1–361 mutant rGATA4 were tested for expression of cardiomyocyte-specific genes myl7 and myh6 (synonyms: Myosin Light Chain 2 and Myosin Heavy Chain α, respectively) at st.34 and for levels of GATA4 protein at st. 10 (lower panel). Note that the effect of this deletion is negligible on endothelial marker aplnr (apelin receptor; synonym- msr) and is less pronounced on hba3 (haemoglobin alpha 3; synonym- αglobin), a blood marker. odc1- loading control (expression of ornithine decarboxylase 1). (C) Nuclear localisation of 1–361 rGATA4 was determined by immunohistochemistry at st. 7–8. (D) Lack of activity of 1–361 *in vivo*. Embryos injected with 1–361 rGATA4 at 8–16 cell-stage in anterior ectodermal precursors were analysed for myl7 expression by whole mount hybridisation. myl7 expression was only observed in the heart (h) in all embryos examined (n = 50, from 2 independent experiments). In contrast, cardiogenic GATA4 versions readily induce ectopic myl7 expression (Fig. 3, Fig. 5). (E) Non-cardiogenic 1–361 GATA4 mutant retains gene expression-inducing activity in animal cap explants. At st. 10 the mutant 1–361 induces endogenous gata4 and (F) at st. 34 pan-endodermal marker alpha-2 macroglobulin (a2m; synonym – endodermin) is induced (as well as by N-terminal deletions 153–440 and 201–440 non-cardiogenic GATA4 (Gallagher et al., 2012)).

We have recently demonstrated that a region within the N-terminal domain of GATA4, aa 129–153, is required for cardiogenesis in pluripotent animal cap explants from *Xenopus* embryos (Gallagher et al., 2012), but the roles of the C-terminal region of GATA4 were not examined. To investigate the role of the C-terminal region in mediating cardiogenic activity of GATA4, we first tested a deletion that removes the final 78 amino acids, rGATA4 1–361. The mutant protein was efficiently produced in embryos and was properly localised to the nuclei, but it was incapable of inducing cardiomyocyte differentiation, both in animal cap explants and in embryos (Fig. 1B). In contrast, the 1–361 mutant has retained the capacity to induce markers of endothelium and blood (Fig. 1).

Further evidence that the 1–361 mutant was not simply inactive, despite being non-cardiogenic, came from extended marker expression analysis: the endogenous gata4 was found to be induced at st. 10 (gastrulation) and a marker of endoderm, a2m (endodermin) was expressed at st. 34 (Fig. 1E,F). These results suggest that the removal of carboxy-terminal 78 amino-acids selectively affects cardiogenic activity of the GATA4 protein. Amino acid sequence of the C-terminus of GATA4 that is required for cardiogenesis is poorly conserved with a related cardiogenic factor GATA5 and offers no obvious clues about its mechanism of action (Fig. 1A).

### 3.2 Carboxy-terminal domain of GATA4 is required for interaction with CDK4

**Fig. 2:**
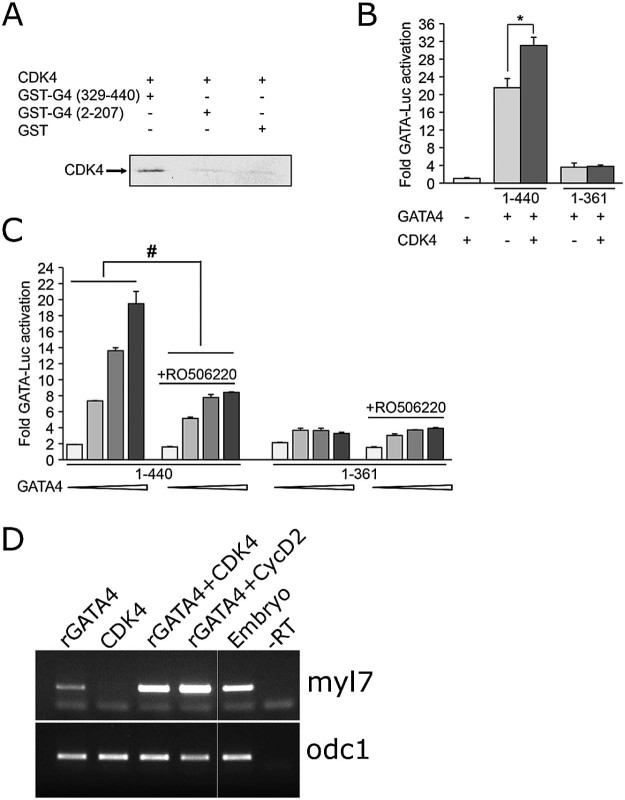
CDK4 interacts with C-terminal GATA4 *in vitro* and enhances cardiogenic activity of GATA4 (A) *In vitro* translated ^35^S radio-labelled CDK4 protein was incubated with GST-N-terminal GATA4, GST-C-terminal GATA4 or GST proteins on glutathione sepharose beads. The bound proteins were resolved by SDS/PAGE and revealed by autoradiography. Note that CDK4 binds C-terminal GATA4 (1st lane). The experiment is one representative of two. (B) Fold synergy of wild type GATA4 (1–440) or C-terminal deletion (1–361) with CDK4 on GATA-dependent promoter. The C-terminal region is required for synergy. 10 ng of GATA4 and 100 ng of CDK4 expression vectors were used. The experiment was performed twice. * p < 0.005 of GATA4 1–440/CDK4 fold synergy vs GATA4 1–440 using Student's t test. (C) NIH3T3 cells were transiently transfected with GATA-luc and increasing doses of the indicated GATA4 expression vector (5, 25, 50 and 100 ng) with our without treatment with 3 µM CDK4 inhibitor. The cells were treated the following day after transfection and kept for 18 hrs. Note that 1–361 GATA4 activation is not inhibited by CDK4. Experiment was performed twice. # p < 0.001 of GATA4 1–440 with CDK4 inhibitor vs. GATA4 1–440 using two-way ANOVA. (D) CDK4 enhances cardiogenic activity of GATA4. Animal cap explants injected with indicated capped mRNAs (400 and 100 pg/embryo of CDK4 and CyclinD2 (CycD2), respectively; 300 pg/embryo of rGATA4 (suboptimal dose)) were analysed for expression of cardiomyocyte marker myl7 at st. 34.

To investigate the mechanisms through which the carboxy terminal domain of GATA4 might operate, using candidate approach we focused on CDK4. In a recent study we have shown that CDK4 activity is required for GATA4-driven transcription (Yamak et al., 2014). One way in which CDK4 could affect GATA4 activity is through enabling synergism between GATA4 and Cyclin D2, as physical and functional interactions of GATA4 with Cyclin D2 depend on S160, a residue that can be phosphorylated by CDK4 *in vitro* (Yamak et al., 2014). To determine which region of GATA4 is required for interaction with CDK4, we performed *in vitro* pull down assays of CDK4 with GST-N- or C-terminal GATA4 proteins. Fig. 2A shows that CDK4 only binds the C-terminal region of GATA4. As stated above, our previous results have indicated that CDK4 phosphorylates N-terminal domain of GATA4 and that amino acid S160 is required for phosphorylation (Yamak et al., 2014). Taken together, these data suggest that CDK4 binds to and phosphorylates distinct domains of GATA4.

We next wished to determine if the C-terminal domain of GATA4 is required for transcriptional synergy between GATA4 and CDK4 (Yamak et al., 2014). To do this, we co-transfected wt GATA4 or C-terminal-deletion mutant 1–361 with CDK4 and a GATA site-dependent reporter. As shown in Fig. 2B, full length (wt) GATA4 but not the C-terminal mutant synergises with CDK4 in transcriptional activation. The requirement of GATA4 C-terminal domain for interaction with CDK4 was confirmed by testing the effect of CDK4 inhibitor on C-terminal mutant. Consistent with our previous data (Yamak et al., 2014), treatment with CDK4 inhibitor reduces GATA4 activation of its target promoter dramatically. This effect requires the C-terminal domain of GATA4 (Fig. 2C).

To test whether transcriptional synergy between GATA4 and CDK4 observed in cell culture might be of relevance to cardiogenic activity of GATA4, we examined the ability of GATA4 to induce cardiac differentiation in animal cap explants in the absence or presence of exogenous CDK4 or CyclinD2, an established cardiogenic cofactor of GATA4 (Yamak et al., 2014). CDK4 alone had no effect on cardiogenesis, but it enhanced the cardiogenic activity of GATA4 similar to CyclinD2 (Fig. 2D), suggesting that transcriptional synergy between GATA4 and CDK4 might have a role in cardiogenesis.

**Fig. 3:**
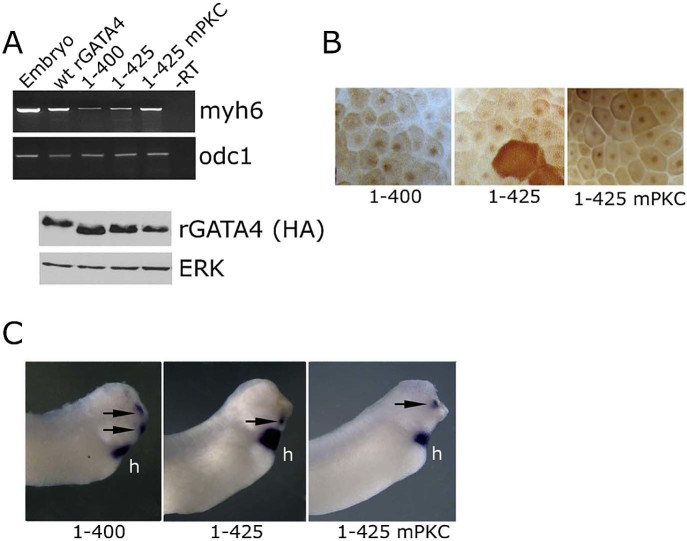
Deletion of 15 or 40 C-terminal amino acids does not block cardiogenic activity of GATA4. (A) Expression of myh6 and odc1 in st. 34 animal caps expressing wt, 1–400, 1–425 and 1–425 mPKC (S419A; S420A) versions of rGATA4. Western blot analysis (lower panel) of protein levels at st. 10. (B) Nuclear localisation of 1–400, 1–425 and 1–425 rGATA4. (C) Embryos injected in anterior ectodermal blastomeres at 8–16 cell stage with 1–400, 1–425 and 1–425 were analysed for myl7 expression at st. 34. myl7 was detected in the heart (h) and in ectopic locations (arrows) of representative embryos (out of 40, 4–7 embryos showed ectopic cardiac expression).

To refine the C-terminal region of GATA4 that is required for cardiogenesis, we tested smaller deletions, 1–425 and 1–400. Both deletions did not abolish cardiogenic activity of GATA4 and had not altered protein stability or nuclear localisation (Fig. 3) thus mapping the C-terminal cardiogenic region to aa 362–400. We have additionally tested point mutations that abolish protein kinase C (PKC) phosphorylation sites (S419A and S420A), as PKC was shown to enhance activity of GATA4 in cell culture (Wang et al., 2005). These mutations (mPKC) had no effect on cardiogenic activity of GATA4, suggesting that PKC modification is not involved in regulation of GATA4-mediated cardiogenesis (Fig. 3).

### 3.3 Partial rescue of cardiogenic activity of inactive rGATA4 mutants by a heterologous activation domain

**Fig. 4:**
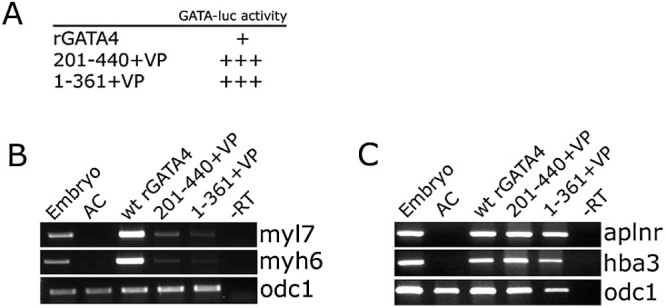
Non-cardiogenic GATA4 mutants lacking N- or C-terminus can be partially rescued by a heterologous transcriptional activator. (A) 1–361 and 201–440 VP fusions are stronger transcriptional activators than the wt rGATA4. Embryos were injected at 1- or 2-cell stage with a firefly luciferase reporter under the control of two GATA sites, *Renilla* luciferase plasmid driven by the thymidine kinase (TK) promoter and indicated mRNAs. Control-DNA alone sample. Animal caps were excised at st. 8.5 and collected for analysis 3 hours later. GATA-luc activity represents relative fold activation (firefly luciferase activity normalised by Renilla luciferase activity, with control (no mRNA) sample set at 1). In three separate experiments 201–440 + VP and 1–361 + VP were found to be considerably more active than the wt rGATA4, with variable fold differences but consistent trend of 201–440 + VP and 1–361 + VP showing 5–10 times greater activity than the wt rGATA4. (B) VP16 minimal activation domain (VP) weakly rescues cardiogenic activity of 1–361 and 201–440, as assessed by expression of myl7 and myh6. (C) In contrast, the ability of 1–361 and 201–440 VP fusions to induce the expression of endothelial marker aplnr and a blood marker hba3 is comparable to the wt rGATA4.

The non-cardiogenic mutant rGATA4 aa 201–440 (Gallagher et al., 2012) lacks the known activation domains in the N-terminus of the protein (Morrisey et al., 1997), and similarly the 1–361 mutant lacks the region required for transcriptional activation (Zhou et al., 2012). These observations raise a possibility that these mutants may be non-cardiogenic simply because of compromised transcriptional activity. An additional question raised by these results is whether the deleted transactivation domains provide ‘generic’ activity or are perhaps specifically required for the cardiogenic role of the protein. To address these questions we supplied two copies of the minimal transactivation domain from strong transactivator VP16 (Brickman et al., 2000) to the 1–361 and 201–440 mutants. The resulting fusion proteins behave as more potent transactivators of a GATA site-driven synthetic reporter gene than the wt rGATA4 (Fig. 4A). In contrast, they are much weaker cardiogenic inducers compared to the wt rGATA4 (Fig. 4B). These results suggest that the N- and C-terminal domains of rGATA4 do not simply have a generic transactivation role but additionally provide a degree of specificity to cardiogenic activity of the protein. This conclusion was supported by examining the expression of markers of other cell fates induced by GATA4: the 201–440 and 1–361 VP fusions were indistinguishable from the wt rGATA4 in their ability to induce the expression of endothelial and blood markers (Fig. 4C).

### 3.4 GATA4-induced cardiogenesis does not require residues important for interaction with FOG2 and Tbx5

**Fig. 5:**
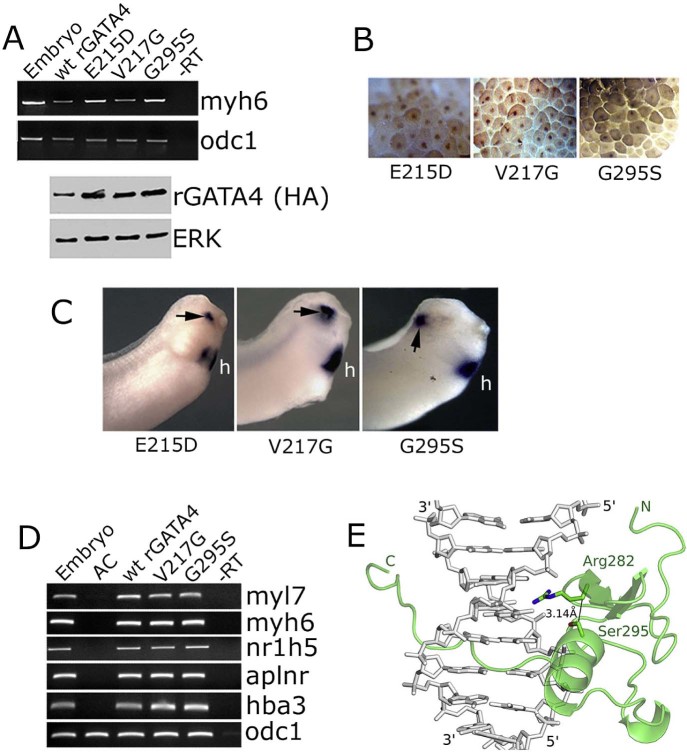
Cardiogenic activity of GATA4 does not require residues which are essential for interaction with FOG2 or Tbx5. (A) E215D, V217G and G295S mutations do not affect cardiogenesis induced by GATA4 in animal pole explants, as assessed by expression of myh6 at st. 34. Bottom panel – Western blot analysis of rGATA4 variants in animal caps collected at st. 10. (B) Nuclear localisation of E215, V217G and G295S GATA4 mutants. (C) Ectopic myl7 expression (arrows) induced by indicated mutants in representative embryos (out of 50, 4–10 embryos showed ectopic myl7 expression). (D) In addition to being able to efficiently induce cardiac differentiation markers myh6 and myl7, V217G and G295S mutants are indistinguishable from the wt rGATA4 in their ability to induce liver (nr1h5; synonym-for1), endothelium (aplnr) and blood (hba3) markers. (E) Model of the C-terminal Zn finger of GATA4 with G295S mutation. The key side chains, Ser295 and Arg282, are indicated. The model shows the clash that would occur with the mutation in the absence of backbone or side chain adaptations to resolve it.

GATA4 has multiple roles in development and in post-embryonic life. It is thought that these roles are at least in part mediated through interaction of GATA4 with various cofactors. One of these factors, FOG2, interacts with the N-terminal Zn finger of GATA4 to mediate development of coronary blood vessels, and V217 is essential for this interaction (Crispino et al., 2001). A mutation of a residue in the vicinity of V217, E215D, has been implicated in cardiac roles of GATA4 as it has been detected in some patients with Tetralogy of Fallot (Nemer et al., 2006). As shown in Fig. 5, V217G and E215D mutations did not affect the ability of rGATA4 to induce cardiac differentiation in animal cap explants.

The C-terminal Zn finger of GATA4 interacts with numerous partners, including T box transcription factor Tbx5, an important regulator of heart development. The importance of the interaction of GATA4 and Tbx5 for heart development has been suggested by human genetics studies which have reported patients with congenital heart disease carrying the G296S mutation in the GATA4 protein (Garg et al., 2003). The G296S mutation was found to affect the interaction of GATA4 with Tbx5, in addition to leading to reduced DNA binding and transactivation activities (Garg et al., 2003).

We next tested the effect of G295S mutation on the cardiogenic activity of rGATA4 (Gly at position 295 in the rat GATA4 is equivalent to Gly 296 in the human protein). The mutant protein was found to have wt-like cardiogenic activity (Fig. 5). In addition, the G295S mutation did not affect the ability of GATA4 to induce markers of endothelium, blood and liver (Fig. 5). This finding was unexpected, in light of reported compromised DNA binding of the G296S mutant (Garg et al., 2003), and we wished to examine it in more detail.

We have established that the rat GATA4 G295S affects DNA binding of the protein (data not shown), in agreement with previous results. To better understand the structural basis for properties of the mutant protein, we considered several possible explanations. First, the glycine residue might adopt a main chain conformation that is inaccessible to amino acids with side chains. Hence, the G295S mutation might enforce changes in main chain conformation and thus destabilise the structure. Second, the serine 295 might directly interfere with DNA binding, due to a steric clash with the DNA. Finally, G295S mutation might indirectly influence the DNA binding properties of GATA4.

In order to distinguish between these possibilities, we first modelled the complex of the second zinc finger of rat GATA4 with target DNA, using the solution structure of DNA-bound GATA1 as the template (pdb-accession 3GAT, Omichinski et al., 1993). We considered this as a valid approach, because GATA1 and GATA4 share the specificity for the GATA sequence in target DNA and are highly similar in the region around conserved G295. We then manually introduced a serine residue in the position of glycine 295. We note that in the template structure and in the model, Gly adopts a helical main chain conformation that is also accessible to serine. Hence, a destabilisation of the GATA4 (and also GATA1) structures due to Ramachandran angle constraints for serine (or other amino acids with side chains) is unlikely. Also, the model shows that the serine is too far away to clash with the DNA directly, eliminating the second possible explanation of the G295 effect on DNA binding. However, we found that the introduced serine 295 in the GATA4 model (and also the GATA1 template) packs too tightly against the side chain of arginine R282, a predicted DNA-contacting side chain (Fig. 5E). The clash is relatively mild (distance from the Cβ of S295 to the arginine side chain ≈ 3.2 Å), but independent of the choice of serine rotamer, since it also involves the Cβ, which has a Cβ position uniquely defined by the main chain conformation. The R282 has hydrogen bonds with the guanidino group of its side chain with the Hoogsteen edge of a guanine of the GATA binding site. Hence, the model is consistent with an indirect effect of the G295S mutation on DNA binding through an effect of the mutation on the conformation of R282. We note the limitations of this explanation for the reduced DNA binding of GATA4 G295S that the clash is only mild and is within the range of coordinate errors for models based on similar templates.

### 3.5 Cell autonomous induction of cardiogenesis by rGATA4

We have shown that *Xenopus* GATA4 induces endoderm in animal cap explants from *Xenopus* embryos (Gallagher et al, 2012, Latinkic et al, 2003) and that it induces cardiogenesis both cell autonomously and non-cell autonomously (Latinkic et al., 2003), suggesting that non-cell autonomous cardiogenesis is mediated by cardiogenic endoderm. As our model for structure–function mapping of rGATA4 depends on exclusive cell autonomous or direct mode of cardiogenesis, we investigated this issue in more detail.

**Fig. 6:**
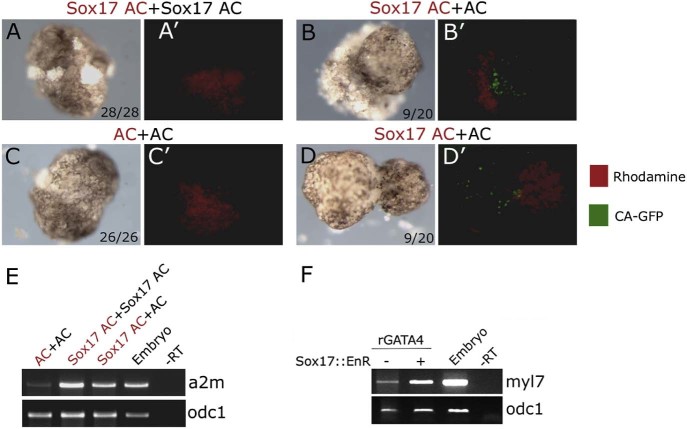
Non-cell autonomous induction of cardiogenesis in animal cap explants by Sox17. Animal caps derived from cardiac actin–GFP (CA-GFP) embryos injected with 200 pg of Sox17 mRNA or uninjected (AC) were conjugated and cultured until st. 34. Different animal caps in each conjugate were distinguished by the presence of rhodamine-dextran in one of them. (A–D) Bright field images of representative examples of indicated types of explants. (A'–D') Corresponding red and green fluorescence images (merged), showing contribution of rhodamine-dextran injected animal caps and the presence or absence of striated muscles (CA-GFP; Latinkic et al., 2003). CA-GFP activity was only detected in naïve, uninjected part of Sox17+AC conjugates, indicating non-cell autonomous cardiac induction. The number of conjugates showing the represented phenotype and the total number of conjugates are shown in the lower right corner (A–D). (E) Activity of Sox17 is shown by induction of endoderm marker a2m in st. 10 animal cap explants. (F) Inhibition of Sox17 function by Sox17::EnR does not block cardiogenesis by rGATA4. rGATA4 (400 pg/embryo) and Sox17::EnR (200 pg per embryo) were uniformly injected at 1- or 2-cell stage and RT-PCR was performed at st. 34 for myl7 and odc1 loading control.

GATA4 induces early endoderm transcription factor, Sox17 (Gallagher et al, 2012, Latinkic et al, 2003). We have tested the ability of Sox17-expressing animal cap explants to induce cardiogenesis when conjugated to Sox17- or un-injected animal caps. Cardiogenesis was only induced by Sox17-expressing animal caps in naïve, uninjected animal caps (Fig. 6). In both types of conjugates (i.e., Sox17 + Sox17 and Sox17 + AC) Sox17 induced endoderm (Fig. 6E). These result demonstrate that Sox17 only induces cardiogenesis indirectly, non-cell autonomously. In our cardiogenesis assays for rGATA4, the mRNA is injected uniformly, making non cell-autonomous mode of cardiogenesis, like the one demonstrated for Sox17, unlikely. To directly test if Sox17 is involved in rGATA4-mediated cardiogenesis, we interfered with Sox17 function by using dominant-negative construct Sox17::EnR (Hudson et al, 1997, Latinkic et al, 2003). Expression of Sox17::EnR caused severely compromised endoderm development, confirming its activity (data not shown). We found that interfering with Sox17 function enhances rGATA4-mediated cardiogenesis (Fig. 6F), showing not only that Sox17 is not required for cardiogenic activity of rGATA4, but also that it might have an inhibitory cell-autonomous effect. Finally, we have used the enhancement of GATA4-mediated cardiogenesis by Sox17::EnR to confirm that inactive rGATA4 mutants, 1–361 and 201–440, do not have residual level of activity (data not shown).

## 4 Discussion

### 4.1 Cell autonomous cardiogenic activity of mammalian GATA4

In addition to cardiovascular cell types (cardiomyocytes, endothelium, smooth muscle) GATA4 induces early endoderm and liver specification in pluripotent cells from *Xenopus* embryos (Latinkic et al., 2003). As anterior endoderm produces cardiogenic signals, it was important to investigate the mode of action of GATA4 in cardiogenesis assay in more detail. Early endoderm determinant Sox17 was shown to be required for cardiogenesis in mouse ES cells cell non-autonomously (Liu et al., 2007). We have previously shown that GATA4 induces Sox17 in animal cap explants from *Xenopus* embryos (Latinkic et al., 2003), and here we have extended this finding by showing that in our assay Sox17 induces cardiac tissue cell non-autonomously, similarly as in ES cells. However, the activity of Sox17 is not required for cardiogenesis mediated by the rat GATA4, suggesting that the model we use likely represents cell-autonomous and direct action of GATA4.

### 4.2 C-terminal domain of GATA4 is required for cardiogenic activity and for interaction with CDK4

We have previously shown that the aa 129–153 region of the rat GATA4 is required for cardiogenesis in animal cap explants from *Xenopus* embryos (Gallagher et al., 2012). This region is also required for interaction of GATA4 with chromatin remodeller BAF60c (Gallagher et al., 2012). In this work we have extended structure–function mapping of GATA4 to the C-terminus. Our results demonstrate that the region composed of aa 362–400 is required for cardiogenesis, as the 1–361 deletion mutant was inactive, whereas the 1–400 mutant was indistinguishable from the wt GATA4.

An insight into the potential mechanism through which the C-terminal domain of GATA4 might act came from an extension of our recent study which has shown that CyclinD2 is a cofactor of GATA4 in cardiogenesis (Yamak et al., 2014). Cyclin D2 binds to the N-terminal region of GATA4, and a residue critical for CyclinD2–GATA4 synergy, S160, was shown to be phosphorylated by CDK4 (Yamak et al., 2014). However, it was not known if CDK4 directly interacts with GATA4. In this study we show that the C-terminal region of GATA4 interacts with CDK4 *in vitro*, and that the region aa 362–440 is required for their transcriptional synergy (Fig. 2). In addition, we have shown that CDK4 can enhance cardiogenic activity of GATA4 (Fig. 2). These findings provide a potential mechanism through which C-terminal region of GATA4 could be involved in cardiogenesis.

Both cardiogenic regions described within GATA4 (362–400 (this work) and 129–152 (Gallagher et al., 2012)) are located within the domains associated with transcriptional activity (Zhou et al., 2012). Here we have shown that a strong heterologous activation domain from VP16 can weakly rescue cardiogenic activity missing in the N- and C-terminal deletions (1–201 and 361–440) of GATA4. The evidence suggesting that transcriptional activation domains are involved in mediating cardiogenic activity of GATA4 is perhaps not surprising. More tellingly, even though fusion of VP16-derived activation domain turns non-cardiogenic GATA4 mutants into strong transactivators, the cardiogenic activity of 1–361-VP16 and 201–440-VP16 is weak. This result suggests that the N- and C-terminal regions of GATA4 operate as transcriptional activators with preference for cardiac tissue. Other findings support the view that non-cardiogenic mutants of GATA4 1–361 and 201–440 are not simply transcriptionally inactive: both mutants can induce endoderm in animal cap explants (Fig. 1, Gallagher et al, 2012), suggesting that cardiac- and endoderm-inducing determinants are separable. These findings provide the foundation for investigation of the mechanisms regulating the activity of cardiogenic domains and how they differ from the mechanisms mediating endoderm-inducing activities.

### 4.3 GATA4-Tbx5 and GATA4-FOG2 interactions are not required for GATA4-induced cardiogenesis

G296 of the human GATA4 (G295 in rodent GATA4) is known to be important for efficient DNA binding and for association with Tbx5 (Garg et al., 2003). In particular, mutation G296S in the human GATA4 has been associated with congenital heart defects, highlighting the importance of intact GATA4 for heart development (Garg et al., 2003).

In the current study we have provided a potential structural basis for lower DNA binding affinity of the G295S mutant. Our modelling suggests that the DNA-contacting arginine residue R282 might be destabilised by the bulkier serine side chain at position 295 instead of glycine. Despite the effect of the G295S mutation on DNA binding of GATA4, we found the mutant to be indistinguishable from the wt GATA4 in its induction of cardiomyocyte, endothelial, blood and liver markers in animal cap explants. Our results indicate that the interaction of GATA4 with Tbx5 is not required for cell fate specification by GATA4, and also suggest that lower affinity for DNA *in vitro* of the mutant does not affect its cardiogenic activity. These results suggest that the intrinsically lower DNA binding capacity of the G295S GATA4 mutant can be compensated *in vivo* in the chromatin context. We do not believe that in our gain of function assay mass action leads to such a compensation, as the level of overexpression is modest (400 pg/embryo) and is just above the suboptimal dose (≈300 pg/embryo).

A recent study has investigated the role of G295 in mouse development by creating GATA4 G295S knock-in homozygous animals (Misra et al., 2012). The observed effects – an embryonic lethality phenotype that was milder than the null embryo phenotype – argue that G295S causes partial loss of function. Some targets of GATA4, namely endodermal and a set of cardiac markers, were not affected by the G295S mutation. Instead, the most striking effect seen in G295S mutants was a defect in myocardial proliferation, which led to the development of hypoplastic myocardium and which likely caused lethality.

These results, showing that early heart development in the mouse embryo is largely unaffected by the G295S mutation, are in good agreement with our findings. The difference is that we have not identified a process (or processes) that require G295 in our work using *Xenopus* embryos. This is likely because early heart development in the frog *Xenopus* is independent of proliferation (Movassagh and Philpott, 2008), in contrast to the mouse heart, suggesting that a major role of G295 within GATA4, and by extension of GATA4-Tbx5 interaction, is specifically in regulation of cardiomyocyte proliferation.

It is surprising that a major functional and likely structural defect caused by the G295S mutation has no effect on the ability of GATA4 to induce cardiac and other cell types in *Xenopus* animal cap explants, and similarly, apparently has no effect on a subset of target genes in the mouse embryo (Misra et al., 2012). Finding out how different targets of GATA4 distinguish between the wt and G295S will improve our knowledge of the mechanism of action of this important regulator of gene expression.

Another well described cofactor of GATA4 is FOG2. Work in transgenic mice has shown that a null mutation of FOG2 and a point mutation of GATA4 that specifically abrogates its interaction with FOG2 (V217G), in homozygous animals create very similar phenotypes (Crispino et al, 2001, Tevosian et al, 2000). These are characterised by the defective development of coronary vessels, resulting in lethality at E12.5 and demonstrating that interaction of GATA4 with FOG2 is important for normal heart development (Crispino et al., 2001). Our results have shown that the V217G mutation has no effect on cell fate specification activity of GATA4 in *Xenopus* animal cap explants. Similarly, the E215D mutation, found in patients with Tetralogy of Fallot (Nemer et al., 2006), had no effect on cardiogenic activity of GATA4.

### 4.4 Conclusion – separable and specific determinants are required for various roles of GATA4 in the heart

Here we have assigned a role to C-terminal region of GATA4 (aa 362–400) in cardiogenesis, and we have provided evidence that the basis for this role likely involves transcriptional activation in cardiac precursors. A potential mechanism through which the C-terminal region of GATA4 promotes cardiogenesis is provided by the findings that CDK4 interacts with it, thereby promoting its transcriptional and cardiogenic activity.

Our results in the present study have shown that the determinants of activity of GATA4 in heart morphogenesis and growth, G295, V217 and E215, are not necessary for its cardiogenic activity. We have previously shown that S105, which is required for interaction with Serum Response Factor and is a key mediator of cardiomyocyte hypertrophy, is similarly not required for cardiogenic activity of GATA4 (Gallagher et al., 2012). Taken together, these findings argue that distinct mechanisms are involved in mediating the roles of GATA4 during different stages of heart development and postnatal homeostasis, and offer an opportunity to investigate how multifunctional transcription factors execute their distinct roles *in vivo*.
